# Ornithine decarboxylase levels in peripheral nerve sheath tumors from NF1 patients

**DOI:** 10.1093/noajnl/vdag208

**Published:** 2026-08-11

**Authors:** Victor A Levin, Sonali Panchabhai, Rasha Alfattal, Gregory N Fuller

**Affiliations:** Department of Neuro-Oncology, The University of Texas M.D. Anderson Cancer Center, Houston (V.A.L., S.P.); Department of Neuro-Oncology, The University of Texas M.D. Anderson Cancer Center, Houston (V.A.L., S.P.); Department of Pathology and Genomic Medicine, Houston Methodist Hospital, Houston (R.A.); Departments of Anatomic Pathology (Neuropathology) and Neuroradiology, The University of Texas M.D. Anderson Cancer Center, Houston (G.N.F.)

**Keywords:** fluorescent microscopy, Neurofibroma, NF1, ODC

## Abstract

**Background:**

The study was conducted to determine the level of ornithine decarboxylase (ODC) activity in several types of peripheral nerve sheath tumors to assess ODC as a potential target for alpha-difluoromethylornithine (DFMO), an irreversible inhibitor of ODC.

**Methods:**

A formalin-fixed paraffin-embedded peripheral nerve sheath tumor tissue microarray was developed in the Department of Anatomic Pathology, MD Anderson Cancer Center. ODC levels were measured using immunofluorescence with a polyclonal antibody to ODC and Alexa-Fluor 647 goat anti-rabbit IgG using previously validated and established methodology.

**Results:**

Nuclear and cytoplasmic ODC levels were assessed to determine surrogate ODC activity based on immunofluorescence from 11 patients with a spectrum of nerve sheath tumors, including malignant peripheral nerve sheath tumors (MPNSTs), plexiform neurofibroma, WHO Grade 1 (NF), atypical neurofibroma neoplasm of uncertain biologic potential (ANNUBP), and schwannoma. ODC activity was less than 5 nmol/30 min/min/ug ODC protein in many MPNST, NF, and schwannomas, but higher than 10 nmol/min/ug protein in ANNUBP tumors and one NF tumor.

**Conclusion:**

Nuclear ODC activity varied across peripheral nerve sheath tumors, with relatively low to moderate levels observed in MPNSTs and higher levels in ANNUBP tumors within the small cohort studied. Since DFMO, an irreversible ODC inhibitor, is more effective in slow-growing grade 2 and 3 gliomas with low ODC levels than in higher malignancy grade 4 glioma with high ODC activity, it is reasonable to assume that MPNSTs and neurofibromas, WHO grade, with relatively low to moderate levels of ODC activity may be suitable candidates for DFMO therapy. However, larger studies are needed to validate ODC activity as a predictive biomarker and to determine the therapeutic efficacy of DFMO in peripheral nerve sheath tumors.

Key Points

1. Nuclear ODC levels vary by peripheral nerve sheath tumor type and grade.
2. Peripheral nerve sheath tumors with low ODC levels may benefit from DFMO chemotherapy.

Importance of the Study

Peripheral nerve sheath tumors in patients with neurofibromatosis type 1 (NF1) vary in malignant potential with variable benefit from surgery and chemotherapy. This manuscript adds to the existing literature by introducing a practical, tissue-based immunofluorescence approach to estimate nuclear ornithine decarboxylase (ODC) activity across a clinically relevant spectrum of tumors, including WHO grade 1 neurofibroma, ANNUBP, MPNST, and schwannoma, using a tissue microarray. Whereas prior work has largely emphasized Ras-pathway biology, this study extends the biomarker-therapy framework to NF1-associated nerve sheath tumors and suggests that ODC activity and levels vary with tumor grade (lowest in MPNST, highest in ANNUBP). By benchmarking ODC ranges against published glioma datasets, the study provides a translational framework for selecting patients most likely to benefit from difluoromethylornithine (DFMO) chemotherapy. Future implications include using ODC as a potential stratification biomarker in the design of DFMO-based trials for NF1-associated peripheral nerve sheath tumors, prioritizing low-ODC, potentially slow-growing lesions, and exploring combination strategies with other pathway-directed agents to improve durability and prevent malignant progression.

Neurofibromatosis type 1 (NF1) is a common autosomal dominant disorder that affects about 1 person in 4000. NF1-affected individuals are prone to the development of benign and malignant tumors that are treated when symptomatic by surgery, radiation therapy, and sometimes by chemotherapy. Typical tumors are plexiform neurofibromas (PNs), malignant peripheral nerve sheath tumors (MPNST), optic pathway glioma, and cerebral glioma. Efforts to develop chemotherapy treatment strategies, while slow, have recently yielded the treatment of PN tumors with selumetinib and mirdametinib to slow progression.^1-6^ Chemotherapeutic strategies evaluated through the Neurofibromatosis Clinical Trials Consortium (NFCTC) include: the phase II STOPN trial of the mTOR inhibitor sirolimus for NF1-associated PNs, NFCTC trials of the mTOR inhibitor everolimus (RAD001), and the multicenter phase II cabozantinib (XL184) trial for NF1-related PNs (NCT02101736), Collectively, these NFCTC efforts illustrate that multiple biologically motivated, pathway-directed approaches beyond MEK inhibition (eg mTOR and multi-kinase/RTK inhibition) have been pursued in NF-associated tumors. Additional efforts to uncover and validate additional potential drugs and drug targets continue.^7^

Molecular and genetic knowledge of NF1 today offers insight into the disease and, potentially, to possible druggable targets. It is known that neurofibromin, the protein product of the NF1 gene, acts as a negative regulator of the Ras proto-oncogene to reduce cell growth.^8^ Furthermore, alternative spliced variants of the GAP-related domain of the NF1 gene (NF1-GRD) can mediate hydrolysis of Ras-bound GTP to GDP, resulting in inactivation of Ras protein. It has been proposed that differential expression of type I and type II NF1-GRD transcripts might be an “on/off” switch that regulates the catalytic activity of the NF1 gene product and regulation of neuronal differentiation and tumorigenesis.^9^^,^^10^ It has also been shown that Ras-GTP levels increased 15-fold in NF1 MPNST and 4-fold in benign NF1 neurofibromas compared to non-NF1 schwannomas; neurofibromin and was not expressed in NF1 MPNST.^11^

NF1‑associated tumor progression follows a stepwise model in which NF1 loss initiates Ras‑driven PNs, CDKN2A/B inactivation enables transition to ANNUBP through cell‑cycle checkpoint escape, and subsequent PRC2 loss with additional genetic and epigenetic alterations drives chromatin reprogramming, lineage plasticity, and malignant transformation to MPNST.^12^^,^^13^

Recent consensus recommendations for NF1-associated peripheral nerve sheath tumors support an integrated histologic and molecular classification framework.^14^ In this framework, tumors previously termed “low-grade MPNST” are proposed to be classified as ANNUBP with increased proliferation, thereby avoiding the use of the term “malignant” for tumors with persistent uncertain biologic potential. Molecularly, *CDKN2A/B* homozygous deletion or inactivation supports classification as ANNUBP in the appropriate histologic context, whereas inactivating alterations involving *SUZ12*, *EED*, or *TP53*, or significant aneuploidy, support classification as MPNST, even in the absence of high-grade histologic features.

Genetic studies in mice carrying linked germ line mutations in NF1 and Trp53 have demonstrated the development of MPNSTs, supporting a cooperative and causal role for Trp53 alterations in MPNST development.^15^ In human tumor samples, none of the benign NF1-associated tumors had CDKN2A/p16 deletions, whereas 3 of 6 MPNSTs appeared to have homozygous CDKN2A/p16 deletions.^16^ In addition, methylation analysis and mutation analysis of CDKN2A/p16 in MPNSTs did not reveal any abnormalities. The authors concluded that malignant transformation of NF1 is associated with loss of p16 expression, which is often secondary to homozygous deletion of the gene.

The activation of the Ras signaling pathway is a hallmark of many tumors associated with NF1, including peripheral nerve sheath tumors. Loss of neurofibromin function leads to increased Ras-GTP levels, which promote downstream signaling events that drive cellular proliferation and survival. While Ras pathway activation in NF1‑associated peripheral nerve sheath tumors has been studied primarily in the context of Ras pathway activation, polyamine metabolism, and epigenetically driven metabolic reprogramming, although the field remains less mature than in gliomas or epithelial cancers. A key downstream metabolic consequence of Ras activation relevant to NF1 tumors is upregulation of polyamine biosynthesis, particularly via ornithine decarboxylase (ODC).^17^^,^^18^ ODC, the rate-limiting enzyme in polyamine biosynthesis, is a key downstream effector of Ras signaling^18-20^ and supports cellular proliferation through the generation of putrescine, spermidine, and spermine, which are required for DNA replication, transcription, chromatin organization, and cell-cycle progression.^21^ In NF1-associated peripheral nerve sheath tumors, loss of neurofibromin results in chronic Ras pathway activation,^22^ providing a biologic basis for increased dependence on polyamine metabolism. These observations suggest that ODC is not only a biomarker of tumor biology but also a potential therapeutic target, as inhibition of ODC with α-difluoromethylornithine (DFMO) can reduce intracellular polyamine pools and suppress proliferation in Ras-driven tumors.

While most direct clinical correlations of ODC activity were established in gliomas, ODC is an established Ras‑responsive enzyme, and polyamine metabolism has been repeatedly implicated as a growth‑supporting pathway in NF1‑driven tumors, including MPNST. These findings provided the rationale for evaluating DFMO (an irreversible ODC inhibitor) in Ras‑driven tumors and for extending interest in ODC biology to NF1‑associated PNS malignancies.

There is little doubt that more effective and earlier interventions are needed for patients with NF1 to lessen the development of low-grade or “benign” tumors, and to reduce the likelihood that gliomas and malignant nerve sheath tumors will develop. Based on our prior studies of DFMO in glioma, we hypothesized that if ODC levels were in the lower range in some nerve sheath tumors, then alpha-difluoromethylornithine (DFMO, eflornithine) might be a viable therapeutic strategy. This hypothesis is based on clinical phase II and phase III studies with DFMO against anaplastic gliomas and glioblastoma, which showed that DFMO efficacy was most pronounced in those patients with slow-growing anaplastic gliomas^23-26^ and who had lower levels of ODC by fluorescence microscopy.^27^^,^^28^ To that end, we used our previously validated technique to measure ODC level in formalin-fixed tumors^28^ and applied it to a tumor tissue microarray of NF1 PNS tumors. The current report will show that many nerve sheath tumors have nuclear ODC levels in a range that suggests that they might respond to DFMO.

## Methods

The authors previously developed a quantitative tissue-based immunofluorescence assay to measure ODC expression in formalin-fixed, paraffin-embedded tissues. The assay employed a purified polyclonal anti-ODC antibody conjugated to Alexa Fluor 647 that was validated using transgenic mice with cardiac-specific ODC overexpression, allowing direct comparison of fluorescence intensity with measured enzymatic ODC activity.^28^^,^^29^ In the current study, formalin-fixed tissue sections underwent antigen retrieval, permeabilization, and incubation with the fluorescently labeled anti-ODC antibody and ODC expression was quantified by fluorescence microscopy using 12-bit grayscale images acquired from formalin-fixed tissue sections stained with an Alexa Fluor 647-conjugated anti-ODC antibody. For assay calibration, transgenic mouse hearts with graded levels of ODC expression were analyzed, and fluorescence intensity was measured by computerized image analysis across multiple microscopic fields. Mean grayscale intensity values were then correlated with independently determined biochemical ODC enzymatic activity measured by the release of radioactive carbon dioxide from radiolabeled ornithine. Linear regression demonstrated a significant relationship between fluorescence intensity and enzymatic activity (R² = 0.71), yielding the calibration equation: ODC activity (nmol/30 min/µg protein) = (ODC fluorescence intensity − 564.8)/22.4. This standard curve enabled semiquantitative conversion of tissue ODC fluorescence intensity into estimated ODC enzymatic activity, facilitating analysis of archived formalin-fixed tumor specimens.^28^

This validated relationship was subsequently applied to formalin-fixed glioma specimens to estimate tumor ODC activity, where increasing ODC expression correlated with increasing tumor grade, supporting its use for retrospective biomarker analyses and evaluation of potential associations between tumor ODC levels and response to difluoromethylornithine (DFMO).^29^

For the data in the current manuscript, we used the same assay for assessment of ODC in Neurofibroma (NF) specimens. Tissue microarrays of NF were created, and these included the same transgenic mouse hearts with graded levels of ODC expression that served as controls to derive a standard curve equation, which enabled semi-quantitative assessment of ODC expression in NF tumors.

### Neurofibroma Tissue Microarray Preparation

The light microscopic criteria for the tumors used in this study were those of the consensus review by Lucas and colleagues.^14^ The tissue microarrays of NF were created in the Department of Pathology at MD Anderson Cancer Center. The Institutional Review Board of the University of Texas MD Anderson Cancer Center approved the creation of the array. The arrays were created from biopsy material for eleven patients; they included MPNSTs, grade 1 NF, atypical neurofibromatosis neoplasm of uncertain biologic potential (ANNUBP), and schwannoma. The patient with schwannoma did not have NF1 but was included for comparison. In some cases, patients have more than one biopsy diagnosis. The tissue microarray was interrogated, and representative regions were selected by a board-certified Neuropathologist (GNF) to obtain the best core. From each biopsy sample 3 cores were placed in the array (ie the cores were punched in triplicate). Since some patients had NFs at multiple sites, biopsy samples were taken from each of these sites. On each array there were also 4 heart samples previously removed from transgenic mice with varying levels of cardiac ODC overexpression^28^^,^^30^ which served as controls. The cardiac tissue on the array was from formalin-fixed tissue blocks taken from mice sacrificed between day 2 and week 2 after birth. Portions of the original heart tissue had been studied by biochemical techniques previously to determine ODC activity (nmol/30min/ug protein) and were used by the authors in prior studies.^30^

### Antibodies

The primary antibody used was a rabbit polyclonal antibody previously developed against ODC (Ab-ODC).^17^ It was purified on an amino-link column (Pierce Chemical; Rockford, IL) to which purified 6× His-ODC had been cross-linked. The concentration of the antibody was 0.5 µg/µL. This antibody was made available to us by David Feith, PhD (Department of Biochemistry, Pennsylvania State University College of Medicine, Hershey, PA) as a 650 µg/1300 µL solution in an Eppendorf tube, which upon arrival was stored at 4 degrees. The secondary antibody to Ab-ODC was an Alexa-Fluor 647 goat anti-rabbit IgG purchased from Molecular Probes (Eugene, Oregon) and had a reported binding efficiency of 5 moles dye/mole protein. This antibody was also stored at 4**°**C.

### Immunohistochemical Staining Protocol

The experimental methodology used in these studies was like that used previously.^28^ Slides with sections of heart and NF tumor were deparaffinized in xylene 5 times and dehydrated with decreasing concentrations of ethanol (100%, 95% and 80%). Slides were then placed in 1× Target Retrieval solution **(**#S169984; DakoCytomation, Copenhagen, Denmark) and steamed for 30 minutes for antigen unmasking. After cooling, samples were permeabilized with 0.2% Triton X-100 and then rinsed with pyrogen-free distilled water and Ca and Mg-free phosphate**-**buffered saline (PBS). Samples were blocked with protein blocking solution (X090930; Dakocytomation) for 1 hour to quench nonspecific binding. Slides were then incubated with 40-µL of primary antibody that was diluted in the ratio of 1:5; the diluent used was the same protein blocking solution used in the earlier step. This incubation was performed in a humidity chamber at 4**°**C for 48 hours. After 48 hours, the slides were again rinsed three times with distilled water and PBS, and then secondary antibody was added to the slide. The secondary antibody was diluted in a ratio of 1:20; the diluent was protein blocking solution, and this slide with secondary antibody was incubated for 1 hour in the humidity chamber at 4**°**C. After one hour, slides were rinsed with PBS thrice and mounted with VECTASHIELD mounting medium (H-1000; Vector labs, Burlingame, CA). Since both the NF1 tumors and heart cores were on the same slide, this allowed binding of ODC antibody to all tissue samples at the same time.

### Fluorescence Microscopy

Images were acquired using an Olympus BX61 fluorescence microscope containing a mercury arc discharge for fluorometric excitation. This microscope has a trinocular observation head coupled to a Hamamatsu ORCA-ER digital charged**-**coupled device camera system. The microscope, camera, and data analysis are operational using a Dell OptiPlex GX260 PC and Image Pro 5.1 software developed by Media Cybernatics (Carlsbad, CA). For the measurement of Ab-ODC-Alexa 647, our approach was to measure optical density in the Cy5 window (Chroma Technology; Rockingham, VT**)** using 12-bit grayscale images obtained at 40× magnification for 100 msec for both the heart and the NF samples. Exposure greater than 100ms lead to saturation of tissue samples, thus, 100 ms was determined to be the optimum exposure time for the images. One of the core sample slides was measured in the Cy5 window at 100 ms using only the secondary antibody without the primary antibody to determine the background intensity and detect any autofluorescence; the background intensity was determined to be 200 gray-scale intensity units, which was subtracted from the intensities of the tissues. All image acquisition was performed on the same day. Lag time between subsequent exposures was 2 minutes.

### Image Analysis

Twelve-bit grayscale images were used for all measurements. For heart samples a pixelated bitmap was created as a single pixel value obtained every 50 pixels using Image Pro 5.1 software. This yielded ∼567-pixel intensity values (ie measurements) per heart field. The analysis was repeated on three microscopic fields per heart core. We used the same 3 transgenic heart samples as were used in a previous study that represented the expected range of ODC activity.^28^

For each tumor sample, we studied a minimum of 6 fields per tumor core (∼18 microscopic fields per patient). The fields chosen were contiguous across the widest horizontal plane. Using Image Pro 5.1 software, a manual tagging approach was applied to each ×40 field. For each cell, its nucleus and cytosol were tagged so that they formed a pair. To precisely locate the nucleus and the cytosol, the H&E image of the core was used as a reference guide. We sought to tag the maximum number of pairs of nucleus and cytosol for 1 field because the NFs were from different locations in different patients, and thus some of the fields that had numerous cells yielded a greater number of tag points, whereas the tumors with bigger cells or fewer cells yielded fewer tag points per field. In general, our approach was to obtain the maximum from each field so that the sum of all represented a fair value for that core. Each tag point was set to measure the average intensity of an area of 3 pixels (0.5 µm^2^).

All the numerical files were kept in a Microsoft Excel file, and basic statistical comparisons were made using Excel software. All plots were prepared using GraphPad Prism 7 software.

### Pathology Verification

Using a feature of Image Pro v.5.1 called “snap measurements,” the tag points were “burned” onto their corresponding field such that tagged locations could later be compared to H&E-stained tumor core slides. A neuropathologist (GNF) independently compared the H&E-stained slides and tagged grayscale template to determine the accuracy of tumor nucleus and cytosol tagging.

## Results

Table 1 is a collation of the nuclear ODC activity, by patient number and histology, for the eleven-patient microarray. The data are presented by individual tumor type and for all tumors of that type for the numbered patient in the table. To understand the magnitude of the study, **N fields** in the table represents the number of individual tumor cells interrogated for each tissue block of the array. For instance, for Patient 1 with a diagnosis of MPNST, we measured all tumor cells in 8 array blocks.

**Table 1.** Collation of nuclear ODC activity levels by patient and histology. The lower-case letters in the diagnosis column signify a different tumor from that patient. That associated letter indicates a different tumor block from that patient. Patient 1 and patient 2 are from the same patient but collected at surgery a year apart. Mean ODC Activity units are nmol/30min/µg protein

Patient number	Diagnosis	*N* fields	ODC Activity, nMole/30 min/µg protein		SD	SEM	Lower 95%	Upper 95%
			**Median**	**Mean**				
1	MPNST-1-a	601	0.05	0.75	1.78	0.07	0.61	0.89
1	MPNST-1-b	315	0.05	0.96	2.00	0.11	0.74	1.18
1	MPNST-1-c	536	0.05	0.86	1.72	0.07	0.71	1.00
1	MPNST-1-d	139	0.05	0.05	0.03	0.00	0.05	0.06
1	MPNST-1-e	582	0.05	1.13	1.76	0.07	0.99	1.28
1	MPNST-1-f	460	0.05	0.35	0.95	0.04	0.27	0.44
1	MPNST-1-g	514	0.57	2.31	3.56	0.16	2.01	2.62
1	MPNST-1-h	724	0.05	0.76	1.64	0.06	0.64	0.88
2	MPNST-2-b	389	10.48	10.86	6.24	0.32	10.23	11.48
3	MPNST-3	244	1.71	4.30	5.50	0.35	3.60	4.99
3	NF-3-a	74	12.16	11.35	8.75	1.02	9.32	13.37
3	NF-3-b	55	0.05	0.14	0.37	0.05	0.04	0.24
3	NF-3-c	98	9.01	8.15	6.86	0.69	6.77	9.52
3	NF-3-d	113	0.05	0.82	1.99	0.19	0.45	1.19
4	NF-4-a	279	14.18	14.48	7.90	0.47	13.55	15.41
5	NF-5-a	227	5.85	5.90	4.96	0.33	5.25	6.55
5	NF-5-b	111	1.70	2.37	2.47	0.23	1.90	2.83
6	NF-6-a	61	0.05	0.36	0.75	0.10	0.16	0.55
6	ANNUBP	358	13.17	13.03	8.52	0.45	12.14	13.92
7	ANNUBP	244	17.03	16.99	12.13	0.78	15.46	18.52
8	NF-8-a	64	3.94	5.07	5.33	0.67	3.74	6.40
8	NF-8-b	99	5.89	6.74	5.94	0.60	5.55	7.92
8	NF-8-c	82	2.93	4.13	4.65	0.51	3.10	5.15
9	NF-9-a	125	7.02	7.42	5.41	0.48	6.47	8.38
10	NF-10-a	306	1.13	2.75	3.58	0.20	2.35	3.15
10	NF-10-b	212	7.47	7.09	5.61	0.39	6.33	7.85
11	Schwannoma	587	2.91	4.54	5.35	0.22	4.11	4.97

### Heart ODC Activity Versus ODC Intensity

**Figure 1.**
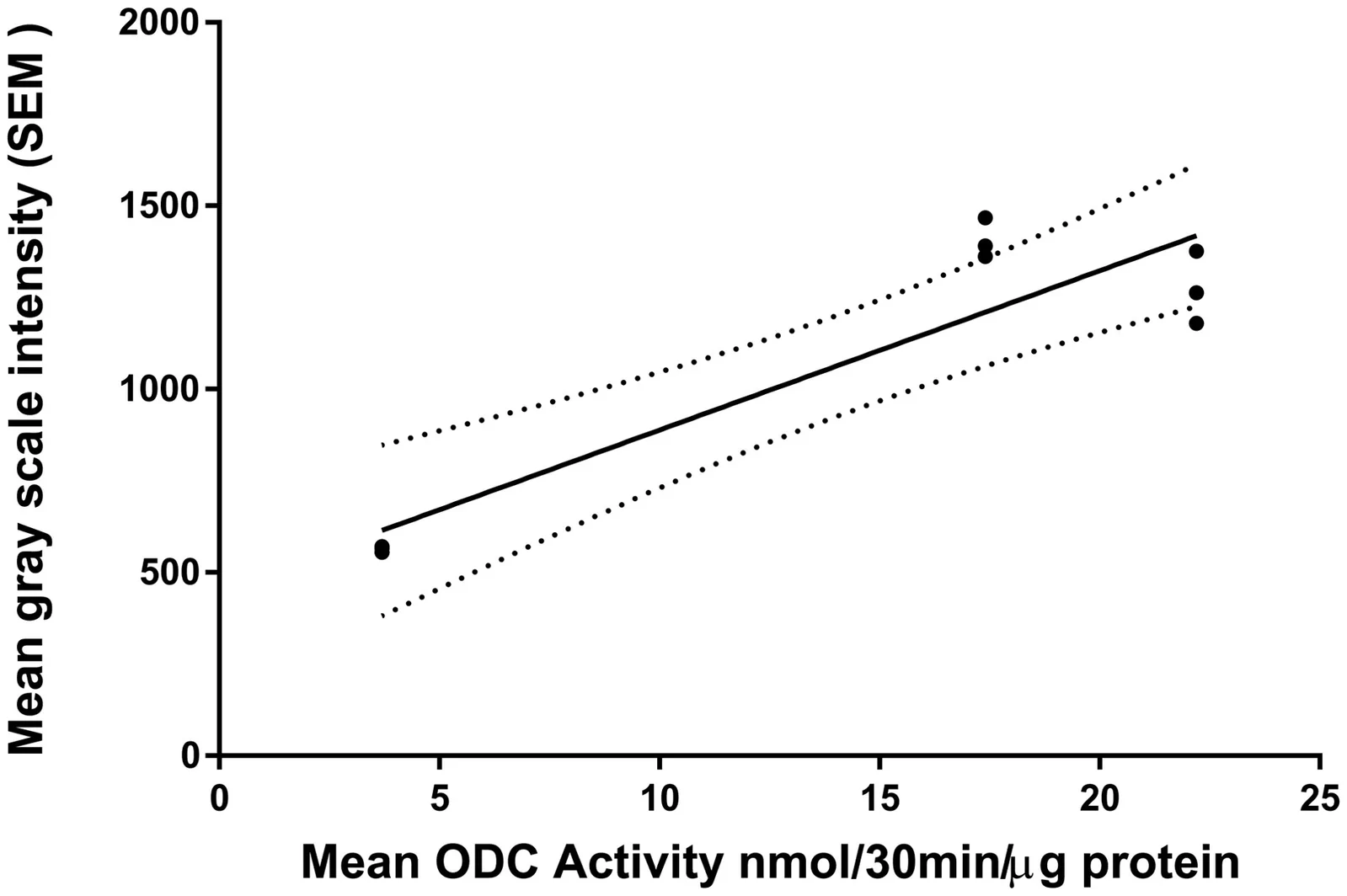
Linear least-squares plot of transgenic heart activity (nmol/30min/ug protein) versus Ab-ODC-Ab-Alexa 647 intensity. The pixilated bitmapped intensity for each of the 3 heart samples in triplicate was used to compute a mean ± SEM. Each heart sample was taken from a different transgenic mouse. Dashed line represents the 95% confidence interval for the regression line that was fitted to the equation Y = 453.7 (± 121.8) + 43.45 (± 7.4) × ODC activity, with R2 = 0.83.

Figure 1 represents the plot of ODC transgenic heart activity (nmol/30min/ug protein) versus ODC-Ab-Alexa 647 intensity based on the pixilated bitmapped intensity for each of the 3 heart samples in triplicate. This methodology used has been used by the author previously.^28^^,^^29^ The least squares regression line was fitted to the equation:


ODC−Ab−Alexa 647 intensity=453.7(±121.8)+43.45(± 7.4)×ODC activity

*R* ^2^ = 0.83 for (Figure 1)

### Tumor ODC Activity

The calculation of ODC activity was based on the rearrangement of the above equation such that: ODC activity (nmol/30 min/µg protein) = (ODC-Ab-Alexa 647 gray-scale intensity 453.7)/43.45. Tumor ODC activity, based on fluorescent intensity measurements of nuclear and cytosolic ODC-Ab-Alexa 647, was computed using the above equation.

**Figure 2.**
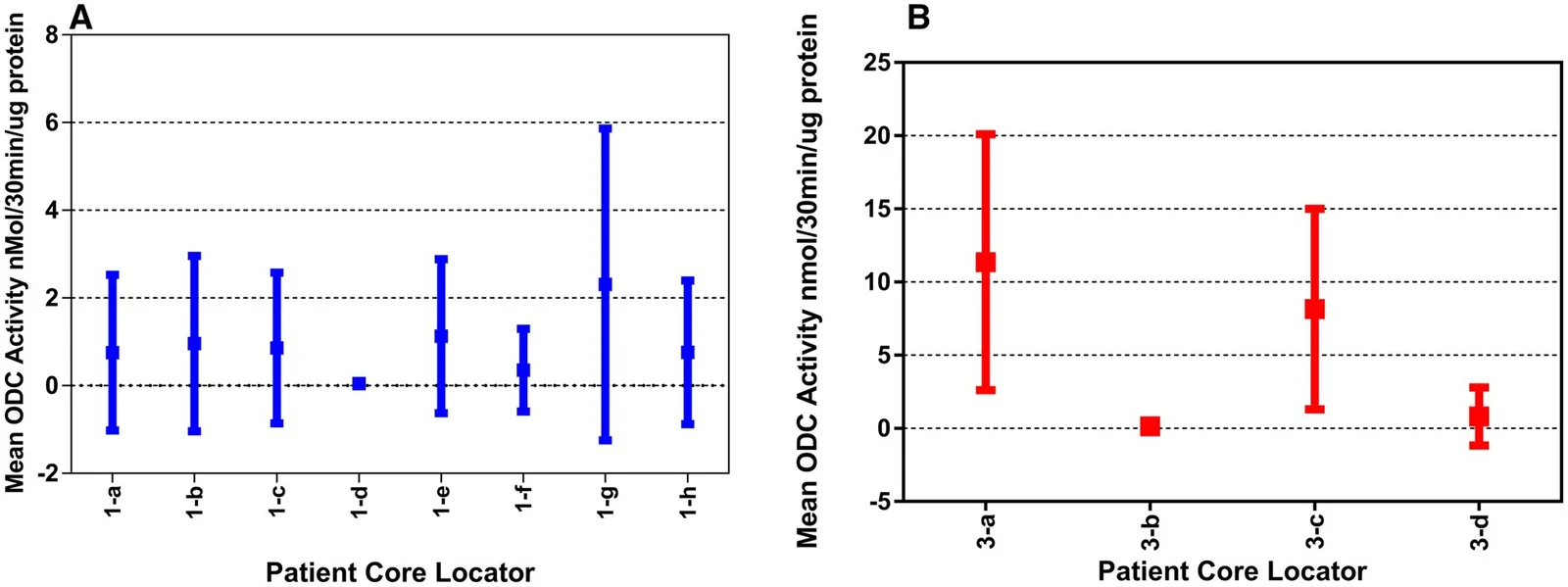
(A) The plot shows the mean +/− standard deviation for the nuclear ODC levels for Patient 1 with MPNST and 8 tissue blocks. (B) The plot shows the mean +/− standard deviation for the nuclear ODC levels for Patient 3 with grade 1 NF and four tissue blocks.

Figure 2A shows the variability in nuclear ODC mean and SD for levels in each of the eight array blocks of Patient 1 with a diagnosis of MPNST. Figure 2B shows the variability in nuclear ODC mean and SD in 4 blocks from Patient 3 with a diagnosis of NF tumor. If the fluorescence intensity was below background, we set ODC activity to the lower end of its sensitivity in our assay, 0.05 nmol/30min/ug protein.

**Figure 3.**
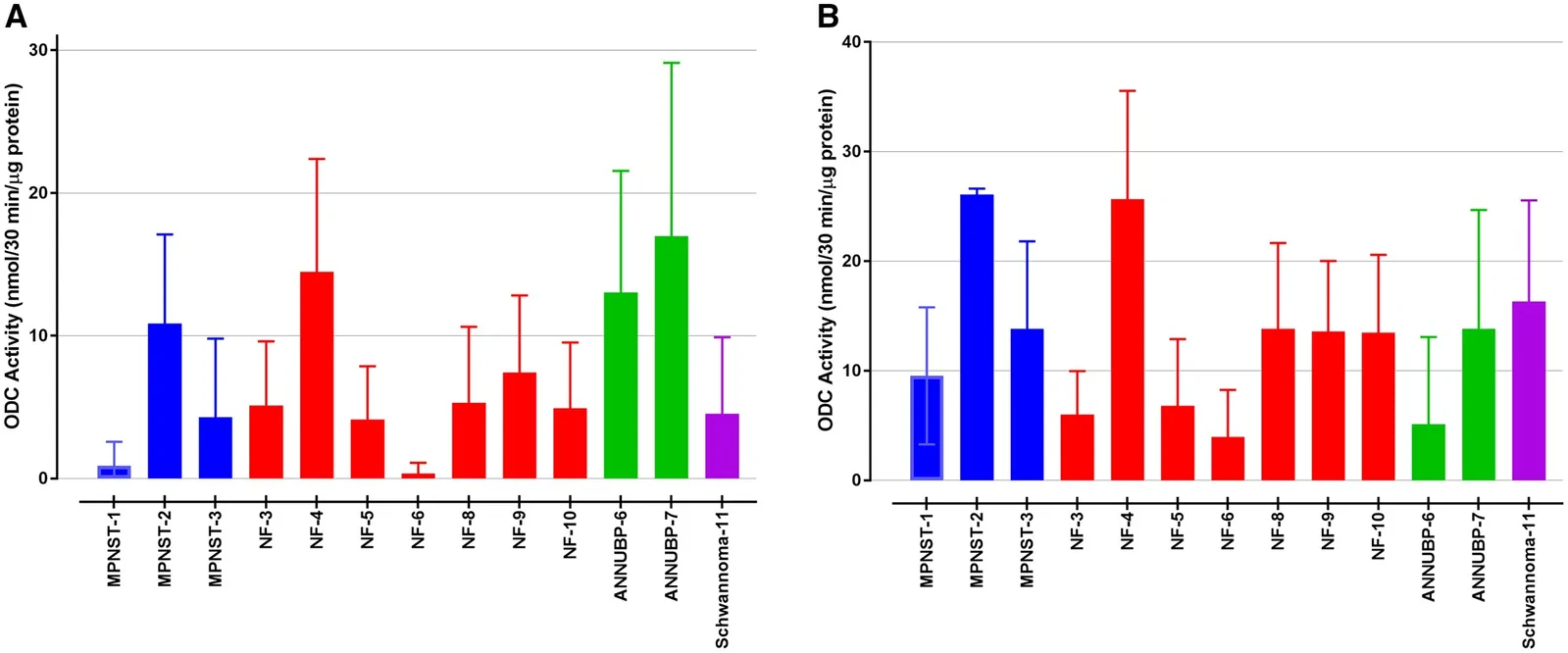
ODC mean activity and standard deviation in the nucleus (A) and cytosol (B) of each patient by tumor. Colum plots are shown with mean +/− SD.

Table 1 is a compilation of nuclear ODC activity with the median, mean, SD, SEM, and upper and lower 95% confidence interval for each tumor core and patient. Figure 2A and 2B are plots of tumor pathology nuclear ODC activity in multiple cores in two patients. Figure 3A is a plot of nuclear ODC activity based on antibody staining of nuclear ODC protein while Figure 3B is a surrogate of ODC activity in the cytosol of the peripheral nerve sheath tumors in this study. Unlike figures 2A and 2B, in these plots each patient’s mean and SD for the entire tumor is plotted. As in the individual plots 2A and 2B, there is a good deal of variability in ODC activity levels between and among tumor types.

## Discussion

The data shown in Figure 3A for surrogate nuclear ODC activity shows that antibody staining of nuclear ODC protein is low to moderate for MPNST tumors, higher for many NF tumors, and highest for the two ANNUBP tumors. The data in Figure 3B for ODC activity in the cytosol, while of interest, does not have the same relevance based on the knowledge we have about ODC activity outside tumor cell nuclei. ODC in the cytosol may be catalytically inactive^31^^,^^32^ or in an inactive complex with antizyme protein.^33^ Polyamines produced in the nucleus through ODC will have a more direct effect on DNA function^34-36^ and methylation of DNA^37^ than polyamines in the cytoplasm that would have to move by diffusion into the nucleus. On the other hand, it is likely that ODC activity in the cytoplasm will impact RNA functionality.^36^^,^^38^

**Figure 4.**
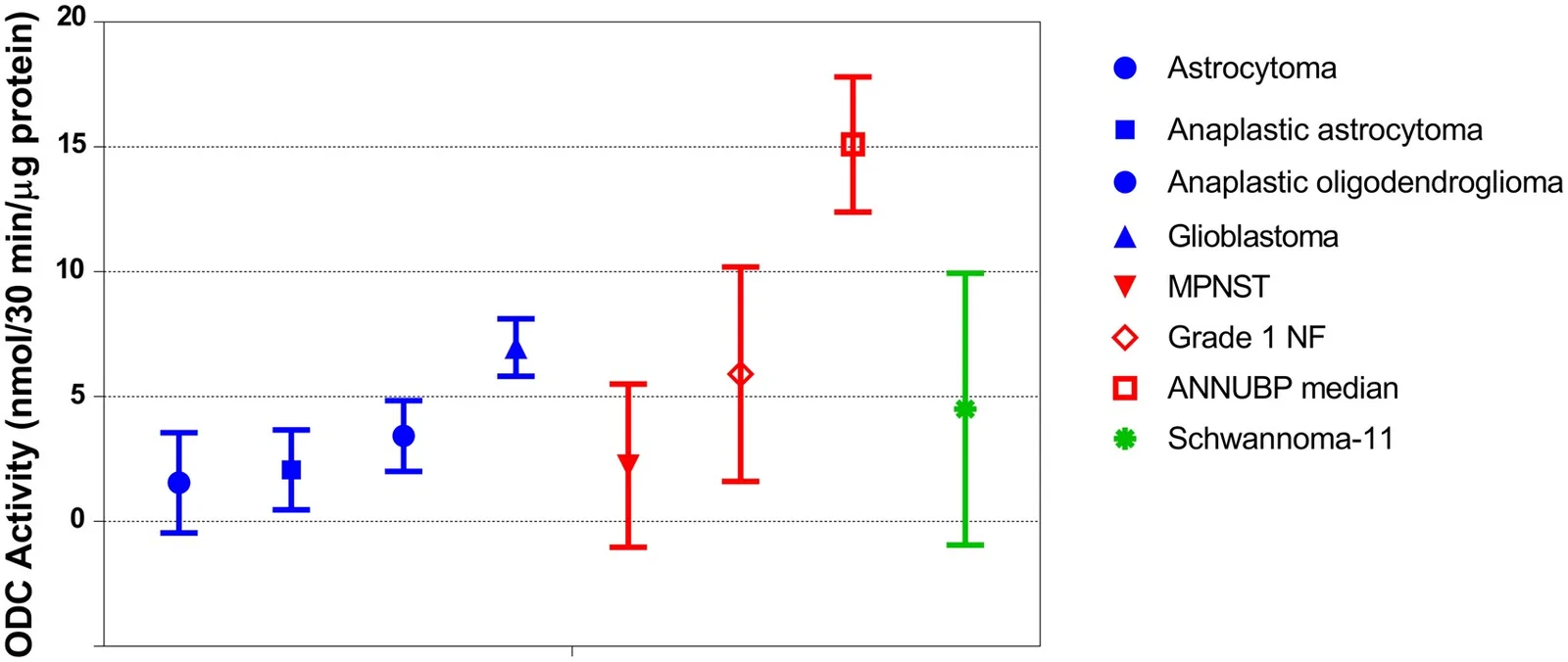
Comparison of published ODC levels for gliomas^28^ compared to those determined in this study. All values are mean +/− standard deviation. Gliomas are shown with blue colored symbols, NF1-associated peripheral nerve sheath tumors have red symbols, the non-NF1-assoiated Schwannoma has a green symbol.

In Table 2 and Figure 4 we compare the observed range of ODC activity measured previously for supratentorial gliomas^28^^,^^29^ to that determined in this study. From this table we could generalize that most MPNST would be likely to benefit from ODC therapy, and only some NF and schwannoma tumors might benefit, but no ANNUBP tumors would benefit. Obviously, these are broad generalizations, but nonetheless, they are based on considerable experience with DFMO (eflornithine) in the treatment of gliomas grades 2-4 and measurements of ODC activity in different glial tumors. In addition, a biological explanation is that tumors with relatively low ODC activity may be operating near the minimum polyamine flux required to sustain proliferation. Because DFMO irreversibly inhibits the rate-limiting enzyme in polyamine biosynthesis,^39^^,^^40^ further suppression of this pathway may reduce intracellular polyamine pools below the threshold necessary for cell-cycle progression.^21^ In contrast, tumors with high ODC activity may possess greater biosynthetic reserve capacity and may therefore be less susceptible to ODC inhibition alone. Additionally, NF1-associated tumors remain driven by Ras-dependent signaling pathways that regulate polyamine metabolism,^19^ suggesting that even tumors with modest baseline ODC activity may remain metabolically dependent on residual ODC function.

**Table 2.** Comparison of ODC activity (nmol/30 min/µg protein) computed based on Ab-ODC-Alexa 647 intensity for glial tumors and tumor types from this study. Glioma ODC means ± SD were computed from the original data sets used for the 2007 publication.^29^ The mean ± SD for groups of each nerve sheath tumor was computed from data of Table 1

Pathology	ODC Activity Mean + SD	Number of tumors	Reference
Astrocytoma	1.55 + 2.0	3	28
Anaplastic astrocytoma	2.1 + 1.6	42	29
Anaplastic oligodendroglioma	3.4 + 1.4	15	29
Anaplastic oligoastrocytoma	1.1 + 0.6	4	29
Glioblastoma	7.0 + 1.2	18	29
MPNST	3.8 + 2.7	3	This study
Grade1 NF	5.9 + 4.3	7	This study
ANNUBP	15.1 + 2.7	2	This study
Schwannoma-1	4.5 + 5.4	1	This study

One possible explanation for the relatively high ODC activity observed in ANNUBP (and one MPNST) is that it is frequently characterized by CDKN2A/B inactivation, which promotes escape from cell-cycle checkpoints and increased proliferation, whereas fully malignant MPNSTs typically acquire additional alterations involving PRC2 components such as SUZ12 and EED, TP53 dysfunction, and broad chromosomal instability. Because ODC is a key rate-limiting enzyme in polyamine biosynthesis and supports proliferative growth, ANNUBP may represent a stage of heightened polyamine dependence during transformation. Subsequent epigenetic and transcriptional reprogramming associated with PRC2-deficient MPNST may reduce reliance on ODC-driven metabolic programs despite greater malignant potential.

Finally, DFMO is primarily cytostatic rather than cytotoxic, raising the possibility that its greatest clinical benefit in NF1 may be in slowing tumor growth and delaying progression rather than inducing tumor regression. In NF1-associated tumors, the goal may not be dramatic regression but rather suppression of proliferation, slowing of tumor growth, reduction in evolutionary pressure for additional mutations, and delayed progression toward ANNUBP or MPNST.

Until recently, there was no approved therapy for adults with nerve sheath tumors, and while there is an FDA-approved therapy for children with PNs, there are challenges with administration and tolerability, so there remains an unmet need among all patients with peripheral nerve sheath tumors. From a therapeutic perspective, our experimental data would encourage a future trial of oral eflornithine, an irreversible inhibitor of ODC, in people with NF1. In addition, oral DFMO can be given in a relatively patient-friendly regime and has an excellent safety profile. It is, therefore, attractive for convenient dosing and, importantly, has potential to improve the quality of life for these patients. We believe that there is significant opportunity for DFMO to impact the treatment landscape of both adult and pediatric patients with NF1. With DFMO now being available for pediatric neuroblastoma patients and showing efficacy in IDH-mutated CDKN2A/B-intact patients,^41^ the safety and tolerability of the drug is well established and warrants the exploration of DFMO for children and adults with peripheral nerve sheath tumors.

## Data Availability

The data described in this manuscript will be made available upon reasonable request via direct communication with the corresponding author.
